# lncRNA TUG1 Facilitates Colorectal Cancer Stem Cell Characteristics and Chemoresistance by Enhancing GATA6 Protein Stability

**DOI:** 10.1155/2021/1075481

**Published:** 2021-11-23

**Authors:** Junfeng Sun, Hangyuan Zhou, Xingqi Bao, Yue Wu, Haowei Jia, Hongchao Zhao, Guanghui Liu

**Affiliations:** Department of Gastrointestinal Surgery, The First Affiliated Hospital of Zhengzhou University, Zhengzhou, China

## Abstract

**Background:**

Chemoresistance and tumor recurrence lead to high deaths in colorectal cancer (CRC) patients. Cancer stem cells (CSCs) contribute to these pathologic properties, but the exact mechanisms are still poorly understood. This study identified that long noncoding RNA (lncRNA) TUG1 was highly expressed in CRC stem cells and investigated its mechanism.

**Methods:**

After the CD133^+^/CD44^+^ cells with cancer stem cell (CSC) characteristics were isolated and identified by flow cytometry, lncRNA TUG1 expression was quantified by quantitative real-time PCR. The lncRNA TUG1 function was further investigated using gain- and loss-of-function assays, sphere formation, Western blot, Cell Counting Kit-8 assay, and cell apoptosis detection. Moreover, the mechanism was explored by RNA pull-down assay, RNA immunoprecipitation, and cycloheximide- (CHX-) chase assays.

**Results:**

lncRNA TUG1 was elevated in CD133^+^/CD44^+^ cells with CSC characteristics. Functionally, lncRNA TUG1 increased the characteristics and oxaliplatin resistance of CRC stem cells. Mechanically, lncRNA TUG1 interacted with GATA6 and positively regulated its protein level and the rescue assays corroborated that lncRNA TUG1 knockdown repressed the characteristics and oxaliplatin resistance of CRC stem cells by decreasing GATA6 and functioned in CRC by targeting the GATA6-BMP signaling pathway. Furthermore, the *in vivo* assay verified the lncRNA TUG1 function in facilitating the characteristics and oxaliplatin resistance of CRC stem cells.

**Conclusion:**

lncRNA TUG1 facilitated CRC stem cell characteristics and chemoresistance by enhancing GATA6 protein stability.

## 1. Introduction

Colorectal cancer (CRC) is one of the most common malignancies worldwide and is a momentous cause of cancer-related death [1]. Although advances have been made in the progression of chemical and targeted therapies, the mortality rates in patients with CRC remain high [2]. Cancer stem cells (CSCs) are tumor-initiating cells in CRC and are responsible for metastasis and recurrence [3, 4]. CSC chemotherapeutic resistance, especially oxaliplatin resistance, is interrelated to CRC recurrence [5]. Thus, investigating the association between the chemotherapy resistance occurrence in CRC and CSCs and revealing the mechanisms are a breakthrough point for CRC treatment.

Long noncoding RNAs (lncRNAs) are RNAs that are more than 200 nucleotides in length and have no protein-coding capabilities [6]. Accumulated studies authenticate that lncRNAs function in multiple biological processes, such as proliferation, metastasis, and chemotherapy resistance of cancer cells [7, 8]. Recently, the pivotal regulatory functions of lncRNAs in maintaining the characteristics and chemoresistance of CRC stem cells have attracted widespread attention. For instance, Zhou et al. illustrated that lncRNA-cCSC1 modulates CSC properties in CRC through activating the Hedgehog signaling pathway, implying that lncRNA-cCSC1 might be a biomarker molecule and promising target for CRC [9]; Ren et al. corroborated that lncRNA H19 exerts as a sponge for miR-141 to facilitate the stemness and chemoresistance of CRC [10].

Taurine upregulated gene 1 (TUG1) is a lncRNA and is originally identified in taurine-treated mouse retinal cells [11]. Studies confirm that lncRNA TUG1 is abnormally expressed in multiple human tumors, mainly containing epithelial ovarian cancer [12], cervical cancer [13], prostate cancer [14], and CRC [15]. Our previous study preliminarily investigates the relationship between lncRNA TUG1 and CRC at the clinical and cellular levels and authenticates that the elevated lncRNA TUG1 facilitates CRC metastasis and is interrelated to the survival time of CRC patients [16]. Although these findings confirm a momentous function of lncRNA TUG1 in carcinogenicity, the lncRNA TUG1 function in the characteristics and chemoresistance of CRC stem cells remains unknown.

In this study, we demonstrated that lncRNA TUG1 was elevated in CRC stem cells and lncRNA TUG1 accelerated the characteristics and chemoresistance of CRC stem cells, and we further investigated the mechanism through various molecular techniques.

## 2. Materials and Methods

### 2.1. Cell Culture

CRC cell lines HCT-116 and SW480 were from American Type Culture Collection (ATCC, Manassas, USA).

HCT-116 and SW480 cells were maintained in Dulbecco's modified Eagle's medium (DMEM) containing 10% fetal bovine serum (FBS, Gibco, Grand Island, USA) and 1% penicillin/streptomycin (Gibco) with 5% CO_2_ at 37°C.

### 2.2. Cell Transfection

pcDNA-NC, pcDNA-TUG1, pcDNA-GATA6, small interfering RNAs against lncRNA TUG1 (si-TUG1), small interfering RNAs against GATA6 (si-GATA6), and negative control (si-NC) were from GenePharma (Shanghai, China).

Cell transfection was carried out as the previously described protocols [17]. The details were as follows: HCT-116, SW480, and CD133^+^/CD44^+^ cells isolated from HCT-116 and SW480 cells and CD133^−^/CD44^−^ cells isolated from SW480 cells were put in 6-well plates for nearly 24 h. The pcDNA-NC, pcDNA-TUG1, si-TUG1, and si-NC were transfected into HCT-116, SW480, and CD133^+^/CD44^+^ cells isolated from HCT-116 and SW480 cells using Lipofectamine 3000 (Thermo Fisher Scientific, Massachusetts, USA); the si-TUG1 was transfected into CD133^+^/CD44^+^ or CD133^−^/CD44^−^ cells isolated from SW480 cells using Lipofectamine 3000; the si-GATA6 was transfected into CD133^−^/CD44^−^ cells isolated from SW480 cells using Lipofectamine 3000; the pcDNA-GATA6 was transfected into CD133^+^/CD44^+^ cells isolated from SW480 cells using Lipofectamine 3000. After nearly 48 h posttransfection, the cells were harvested and the cell transfection efficiency was tested using qRT-PCR and Western blot.

### 2.3. Cell Different Treatments

To evaluate the impact of lncRNA TUG1 on the resistance of CD133^+^/CD44^+^ cells isolated from HCT-116 and SW480 cells to oxaliplatin, the cells silencing or overexpressing lncRNA TUG1 were treated with 0, 1, 2, 4, 8, and 16 *μ*M oxaliplatin (#HY-17371, MedChemExpress, New Jersey, USA) for approximately 48 h.

HCT-116 and SW480 cells silencing or overexpressing lncRNA TUG1 were treated with 4 *μ*M oxaliplatin for 48 h; SW480 cells transfected with si-TUG1 and/or pcDNA-GATA6 were treated with 4 *μ*M oxaliplatin for approximately 48 h.

### 2.4. Flow Cytometry

The CD133^+^/CD44^+^ cells with CSC characteristics from CRC cells were isolated and identified using flow cytometry. HCT-116 and SW480 cells were harvested, followed by the incubation with anti-CD133 (Abcam, 1 : 100, ab216323) and anti-CD44 (Abcam, 1 : 40, ab189524) for nearly one hour. Afterward, the cells were incubated with fluorescein isothiocyanate- (FITC-) labeled secondary antibodies for about 30 min. Subsequently, the cells were resuspended in 100 *μ*l fluorescence-activated cell sorting (FACS) buffer and the stained cells were assessed using flow cytometry (Thermo Fisher Scientific).

### 2.5. Quantitative Real-Time PCR (qRT-PCR)

qRT-PCR assay was carried out as the previously described methods [18]. Total RNA was extracted using Trizol Reagent (Thermo Fisher Scientific). For the analysis of lncRNA TUG1, ALDH1, Nanog, GATA6, and LRH-1, a BeyoRT™ II cDNA Synthesis Kit (Beyotime Biotechnology, Shanghai, China) was conducted to synthesize cDNA from total RNA. Then, a real-time PCR was run on the 7500 real-time PCR system (Applied Biosystems, Thermo Fisher Scientific) with SYBR Green Master Mix (Thermo Fisher Scientific). GAPDH was employed as endogenous controls. Then, the relative expression was tested using the 2^−*ΔΔ*Ct^ method.

### 2.6. Sphere Formation Assay

HTC-116 and SW480 cells and CD133^+^/CD44^+^ cells isolated from SW480 cells (500 cells) were inoculated in 96-well plates, and then, the serum-free DMEM/F12 (Gibco, Carlsbad, CA, USA) with 2% B27 (Gibco), 20 ng/ml EGF (Gibco), and 20 ng/ml basic FGF (Gibco) was added to each well. The number of spheres was evaluated under a microscope (Olympus, Tokyo, Japan).

### 2.7. Western Blot

CRC cells SW480, HCT-116, and CD133^+^/CD44^+^ or CD133^−^/CD44^−^ cells isolated from SW480 cells and CRC tissues were harvested and then were lysed on ice using a RIPA lysis buffer (Beyotime Biotechnology, Shanghai, China). After the concentrations of proteins were quantified using a BCA protein assay kit (Beyotime Biotechnology), the same amount of proteins was separated with sodium dodecyl sulfate-polyacrylamide gel electrophoresis (Thermo Fisher Scientific) and then transferred into polyvinylidene difluoride (PVDF) membranes (Millipore, Billerica, USA). The above membranes were then blocked with 10% skimmed milk for about 1 h, followed by incubating with the antibodies: anti-ALDH1 (1 : 500, Hushi Pharmaceutical Technology), anti-Nanog (1 : 1000, Abcam), anti-GAPDH (1: 500, Abcam), anti-ETV4 (1 : 1000, Invitrogen), anti-ASCL2 (0.2-1 *μ*g/ml, Invitrogen), anti-GATA6 (1 : 1000, Abcam), anti-SOX9 (1 : 1000, Abcam), anti-BMP4 (1 : 10000, Abcam), and anti-BMP2 (1 : 1000, Abcam) overnight at 4°C. After washing, the membranes were then incubated with the secondary antibody (1 : 2000, Abcam) for nearly 1 h at room temperature (RT). The signals of protein bands were visualized using the enhanced chemiluminescence (Thermo Fisher Scientific).

### 2.8. Cell Counting Kit-8 (CCK-8) Assay

The proliferative capacities of CD133^+^/CD44^+^ cells isolated from HCT-116 and SW480 cells were evaluated using a CCK-8 assay (Beyotime Biotechnology). The cells with different treatments were grown in 96-well plates. Then, 10 *μ*l CCK-8 solution was added and continued to incubate at 37°C for nearly 2 h. The optical density was further detected at 450 nm by a microplate reader (Thermo Fisher Scientific).

### 2.9. Analysis of Cell Apoptosis

CD133^+^/CD44^+^ cells isolated from HCT-116 and SW480 cells were collected, and a flow cytometry assay was carried out to assess the cell apoptosis. After the staining with fluorescein isothiocyanate- (FITC-) annexin V and propidium iodide was conducted using a FITC Annexin V Apoptosis Detection Kit (Solarbio, Beijing, China) as the reagent manufacturer's standard procedure, the cell apoptosis was assessed using flow cytometry (Thermo Fisher Scientific) and the above cells were divided into viable cells, dead cells, early apoptotic cells, and apoptotic cells.

### 2.10. RNA Pull-Down Assay

A Magnetic RNA Protein Pull-Down Kit (Thermo Fisher Scientific) was conducted to verify the interaction between lncRNA TUG1 and ETV4, ASCL2, GATA6, and SOX9. Specifically, the biotinylated lncRNA TUG1 was synthesized from RiboBio (Guangzhou, China). CRC cell lysates were incubated with the biotinylated lncRNA TUG1 at 4°C for approximately 1 h. After that, the streptavidin agarose beads were added to the cell protein lysates to precipitate the RNA protein complexes. After washing, the ETV4, ASCL2, GATA6, and SOX9 protein levels in the eluent were tested using Western blot.

### 2.11. RNA Immunoprecipitation Analysis

The interaction between lncRNA TUG1 and GATA6 was confirmed by EZ Magna RNA Immunoprecipitation Kit (Millipore) given the reagent manufacturer's procedures. CRC cell lysates were incubated with anti-IgG (1 : 1000, Abcam) or anti-GATA6 (1 : 1000, Abcam) at RT for about 30 min. Then, the cell lysates were immunoprecipitated with Protein A/G beads at 4°C for nearly 6 h. The immunoprecipitated lncRNA TUG1 was purified and assessed using qRT-PCR.

### 2.12. Cycloheximide- (CHX-) Chase Assay

To assess the lncRNA TUG1 impact on the GATA6 protein degradation, si-TUG1 was transfected into CD133^−^/CD44^−^ or CD133^+^/CD44^+^ isolated from SW480 cells, and the cells were treated with 100 *μ*g/ml cycloheximide (CHX, #HY-12320, MedChemExpress) for 0, 4, and 8 h.

### 2.13. *In Vivo* Tumor Xenograft Model

Twenty-four mice (5-week-old, male) were from Jiangsu ALF Biotechnology Co., LTD. (Nanjing, China). The tumor xenograft model was established as the previously described methods with minor revisions [10]. In particular, CD133^+^/CD44^+^ isolated from HCT-116 cells transfected with Lv-shTUG1 (5 × 10^6^) were subcutaneously inoculated into mice to establish a xenograft model. The mice were injected with oxaliplatin (5 mg/kg) every three days when the subcutaneous graft tumor first appeared and lasted for one week. The mice were sacrificed, and the tumors were removed for subsequent analysis. The tumor volumes were tested by manual calipers and quantified using the formula *V* = 1/2 × larger diameter × (smaller diameter)^2^. All animal assay protocols were approved by the Animal Care and Use Committee of the First Affiliated Hospital of Zhengzhou University.

### 2.14. TUNEL Assay

The apoptosis in the xenograft tissues was determined using a TUNEL Assay Kit (Abcam, Cambridge, UK) as the reagent manufacturer's protocol. All images were obtained and assessed using the microscope (Olympus) and Image-Pro Plus 5.1 software. To determine the percentage of TUNEL-positive cells, five fields were randomly selected under a microscope.

### 2.15. Statistical Analysis

All statistical analyses were evaluated using SPSS 17.0 software. The results were obtained from three independent assays and presented as mean ± standard deviation. One-way analysis of variance (ANOVA) or Student's *t*-test was applied for different analyses. When *P* < 0.05, the differences were statistically significant.

## 3. Results

### 3.1. lncRNA TUG1 Maintains the Characteristics of CRC Stem Cells

**Figure 1:**
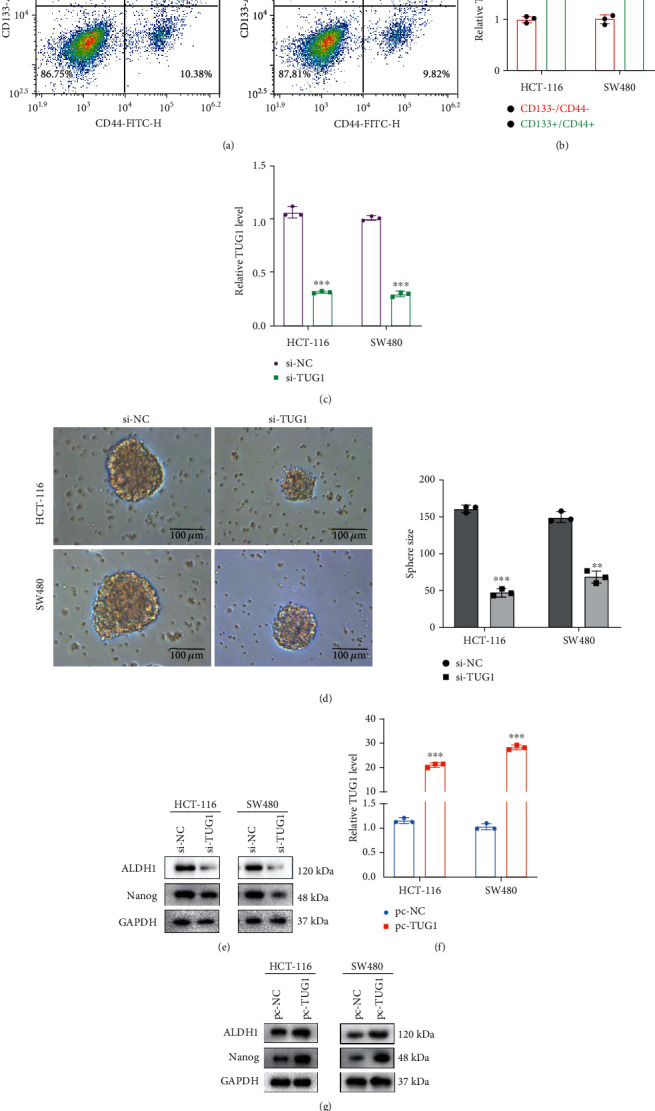
Regulation of lncRNA TUG1 on the characteristics of CRC stem cells. (a) Flow cytometry analysis of CD133^+^/CD44^+^ and CD133^−^/CD44^−^ cells sorted from colorectal cancer (CRC) cell lines HCT-116 and SW480. (b) Quantitative real-time PCR (qRT-PCR) was carried out to quantify the lncRNA TUG1 expression in CD133^+^/CD44^+^ and CD133^−^/CD44^−^ cells. (c) si-TUG1 and its control were transfected into SW480 and HCT-116 cells. The expression of lncRNA TUG1 was assessed by qRT-PCR. (d) The formation of clonal spheres was assessed by sphere formation assay (scale bar: 100 *μ*m). (e) The protein levels of ALDH1 and Nanog in SW480 and HCT-116 cells were measured by Western blot. (f) pcDNA-TUG1 was transfected into SW480 and HCT-116 cells. qRT-PCR was conducted to quantify the lncRNA TUG1 expression. (g) The protein levels of ALDH1 and Nanog in SW480 and HCT-116 cells were detected using Western blot analysis. ^∗∗^*P* < 0.01 vs. CD133^−^/CD44^−^. ^∗∗∗^*P* < 0.001 vs. CD133^−^/CD44^−^, pc-NC, or si-NC. NC: negative control.

To determine whether lncRNA TUG1 is interrelated to CRC stem cells, we isolated and identified CD133^+^/CD44^+^ cells with CSC characteristics from CRC cells HCT-116 and SW480 by flow cytometry. As exhibited in Figure 1(a), CD133^+^/CD44^+^ was expressed in both HCT-116 and SW480 cells, 1.81% in HCT-116 cells and 1.56% in SW480 cells. Meanwhile, lncRNA TUG1 expression was higher in CD133^+^/CD44^+^ than in CD133^−^/CD44^−^ cells (Figure 1(b)).

CSCs proliferate in a spherical form when cultured under nonadherent conditions [19]. Subsequently, we investigated whether lncRNA TUG1 influenced the clonogenic potential of CRC. lncRNA TUG1 was silenced in HCT-116 and SW480 cells, and the transfection efficiency was assessed using qRT-PCR (Figure 1(c)). As displayed in Figure 1(d), silencing lncRNA TUG1 restrained the formation of clonal spheres. Nanog and ALDH1 are pivotal CSC-related molecules [20]. We further investigated the Nanog and ALDH1 protein levels in SW480 and HCT-116 cells and authenticated that ALDH1 and Nanog were decreased in SW480 and HCT-116 cells silencing lncRNA TUG1 (Figure 1(e)). Meanwhile, lncRNA TUG1 was overexpressed in HCT-116 and SW480 cells and the transfection efficiency was confirmed (Figure 1(f)). Western blot analysis demonstrated that Nanog and ALDH1 were elevated in SW480 and HCT-116 cells overexpressing lncRNA TUG1 (Figure 1(g)). The above results collectively corroborated that lncRNA TUG1 maintained the CRC stem cell characteristics.

### 3.2. lncRNA TUG1 Enhances Oxaliplatin Resistance of CRC Stem Cells

**Figure 2:**
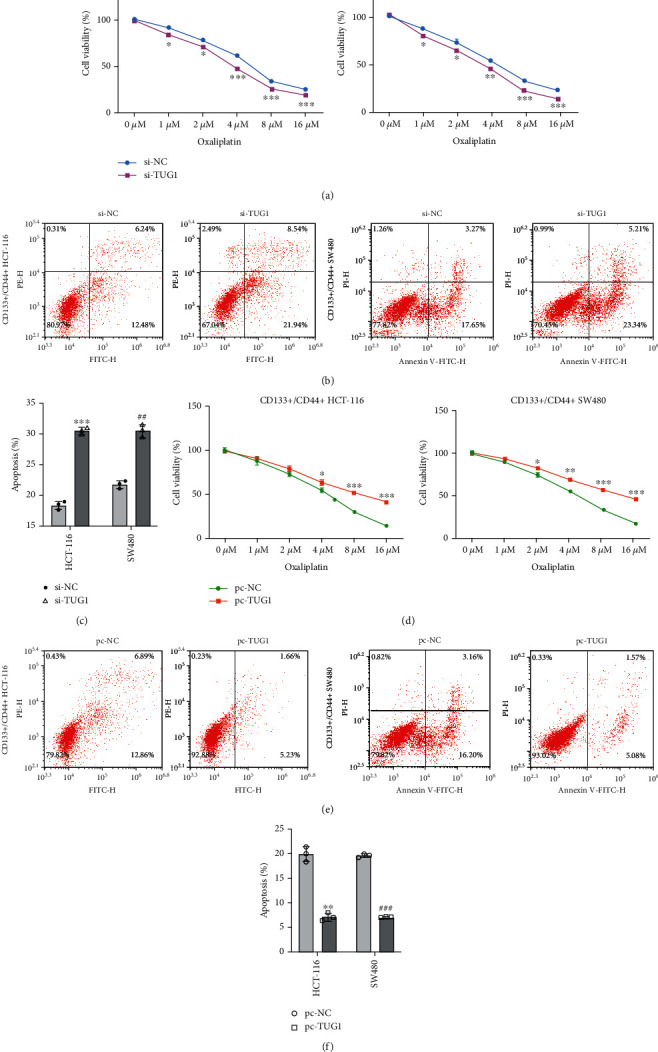
Effect of lncRNA TUG1 on oxaliplatin resistance of CRC stem cells. (a) lncRNA TUG1 was silenced in the CD133^+^/CD44^+^ cells isolated from HCT-116 and SW480 cells, and then, the cells were treated with 0, 1, 2, 4, 8, and 16 *μ*M oxaliplatin for 48 h. Cell Counting Kit-8 (CCK-8) assay was performed to assess the viability of CD133^+^/CD44^+^ isolated from HCT-116 and SW480 cells. (b) lncRNA TUG1 was silenced in the CD133^+^/CD44^+^ cells isolated from HCT-116 and SW480 cells, and then, the cells were treated with 4 *μ*M oxaliplatin for 48 h. The apoptosis of CD133^+^/CD44^+^ isolated from HCT-116 and SW480 cells was detected using flow cytometry. (c) The quantitative results of cell apoptosis. (d) lncRNA TUG1 was overexpressed in the CD133^+^/CD44^+^ cells isolated from HCT-116 and SW480 cells, and then, the cells were treated with 0, 1, 2, 4, 8, and 16 *μ*M oxaliplatin for 48 h. Analysis of the viability of CD133^+^/CD44^+^ isolated from HCT-116 and SW480 cells by CCK-8 assay. (e) lncRNA TUG1 was overexpressed in the CD133^+^/CD44^+^ cells isolated from HCT-116 and SW480 cells, and then, the cells were treated with 4 *μ*M oxaliplatin for 48 h. Detection of the apoptosis of CD133^+^/CD44^+^ isolated from HCT-116 and SW480 cells using flow cytometry. (f) The quantitative analysis of cell apoptosis. ^∗^*P* < 0.05, ^∗∗^*P* < 0.01, and ^∗∗∗^*P* < 0.001 vs. si-NC or pc-NC.

Oxaliplatin is a commonly used chemical treatment for CRC, and its drug resistance seriously restricts its therapeutic effect [21]. To evaluate whether lncRNA TUG1 influenced the resistance of oxaliplatin in CRC, the lncRNA TUG1 was silenced in CD133^+^/CD44^+^ cells isolated from HCT-116 and SW480 cells, and then, the cells were treated with different concentrations of oxaliplatin. As presented in Figure 2(a), the lncRNA TUG1 knockdown weakened the viability of CD133^+^/CD44^+^ isolated from HCT-116 and SW480 cells. On the contrary, the lncRNA TUG1 silence facilitated the apoptosis of CD133^+^/CD44^+^ isolated from HCT-116 and SW480 cells (Figure 2(b)) and the quantitative results of cell apoptosis are displayed in Figure 2(c). Meanwhile, the lncRNA TUG1 was overexpressed in CD133^+^/CD44^+^ cells isolated from HCT-116 and SW480 cells, and then, the cells were treated with different concentrations of oxaliplatin. CCK-8 assay expounded that the lncRNA TUG1 overexpression enhanced the viability of CD133^+^/CD44^+^ isolated from HCT-116 and SW480 cells (Figure 2(d)). On the contrary, the apoptosis of CD133^+^/CD44^+^ isolated from HCT-116 and SW480 cells was repressed after the lncRNA TUG1 overexpression (Figure 2(e)). The quantitative results of cell apoptosis are displayed in Figure 2(f). In summary, lncRNA TUG1 enhanced the oxaliplatin resistance of CRC stem cells.

### 3.3. lncRNA TUG1 Interacts with GATA6 and Positively Regulates Its Protein Levels

**Figure 3:**
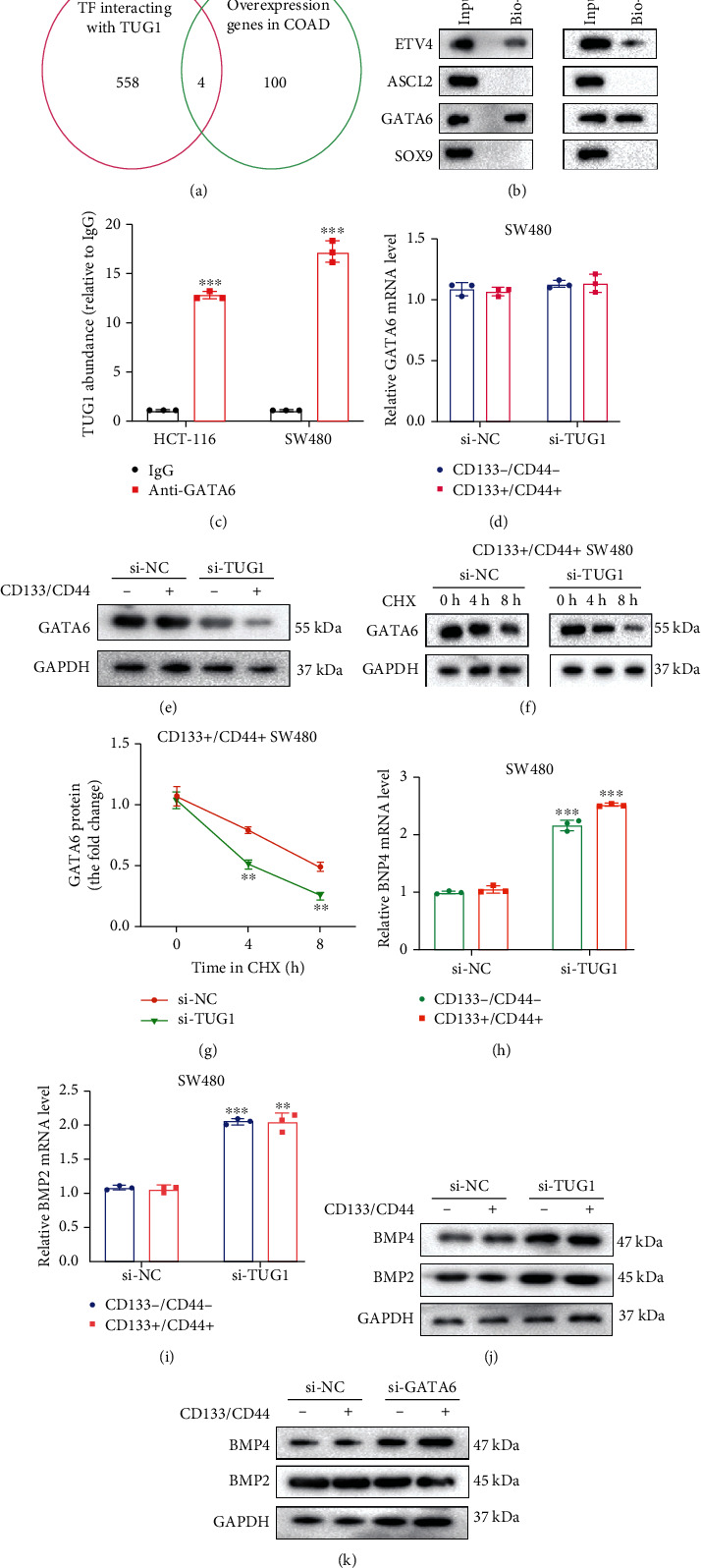
Analysis of the interaction between lncRNA TUG1 and GATA6. (a) RNA Interactome Database and COAD colon cancer database were applied to predict the transcription factors that had potential interactions with lncRNA TUG1 and were highly expressed in CRC. (b) RNA pull-down assay was conducted to verify the interaction between lncRNA TUG1 and ETV4, ASCL2, GATA6, and SOX9. (c) Verification of the interaction between lncRNA TUG1 and GATA6 by RNA immunoprecipitation assay. (d, e) si-TUG1 was transfected into CD133^−^/CD44^−^ or CD133^+^/CD44^+^ isolated from SW480 cells. The mRNA and protein levels of GATA6 were analyzed using qRT-PCR and Western blot. (f, g) si-TUG1 was transfected into CD133^−^/CD44^−^ or CD133^+^/CD44^+^ isolated from SW480 cells, and the cells were treated with 100 *μ*g/ml cycloheximide (CHX) for 0, 4, and 8 h. Detection of the GATA6 protein level by Western blot. (h–k) si-TUG1 was transfected into CD133^−^/CD44^−^ or CD133^+^/CD44^+^ isolated from SW480 cells. The mRNA and protein levels of BMP4 and BMP2 were assessed using qRT-PCR and Western blot. ^∗∗^*P* < 0.01 vs. si-NC or CD133^−^/CD44^−^. ^∗∗∗^*P* < 0.001 vs. IgG or CD133^−^/CD44^−^.

After confirming the impact of lncRNA TUG1 on the characteristics and chemoresistance of CRC stem cells, we further probed into the mechanism. Firstly, we obtained 558 transcription factors that had potential interactions with lncRNA TUG1 through the RNA Interactome Database and obtained the top 100 elevated genes in the COAD colon cancer database (GEPIA2). After taking the intersection, we obtained four candidate molecules ETV4, ASCL2, GATA6, and SOX9 (Figure 3(a)). Meanwhile, RNA pull-down assay was conducted to verify the interaction of ETV4, ASCL2, GATA6, and SOX9 and lncRNA TUG1 in HCT-116 and SW480 cells and the results corroborated that GATA6 was most enriched in the lncRNA TUG1 pull-down complexes (Figure 3(b)). Thus, GATA6 was selected as our main follow-up research molecule. Moreover, the RIP assay further confirmed the interaction between lncRNA TUG1 and GATA6 in HCT-116 and SW480 cells (Figure 3(c)).

Subsequently, we continued to explore the specific mechanism of lncRNA TUG1 regulating GATA6. si-TUG1 was transfected into CD133^−^/CD44^−^ or CD133^+^/CD44^+^ isolated from SW480 cells. qRT-PCR analysis confirmed that there were no notable changes in the GATA6 mRNA level in cells (Figure 3(d)), while the GATA6 protein level was decreased after transfecting si-TUG1 (Figure 3(e)). Cycloheximide (CHX) is a commonly used protein synthesis inhibitor [22]. As displayed in Figures 3(f) and 3(g), the interference with lncRNA TUG1 accelerated the GATA6 protein degradation. Previous studies have shown that BMP4 and BMP2 are the downstream regulatory targets of GATA6 [23, 24]. As exhibited in Figures 3(h)–3(k), the mRNA and protein levels of BMP4 and BMP2 were elevated after transfecting si-TUG1. These data demonstrated that lncRNA TUG1 bound to GATA6 and positively regulated its protein levels and might function in CRC by targeting the GATA6-BMP signaling pathway.

### 3.4. GATA6 Overexpression Attenuates the Inhibitory Effect of lncRNA TUG1 Knockdown on the Characteristics and Chemoresistance of CRC Stem Cells

**Figure 4:**
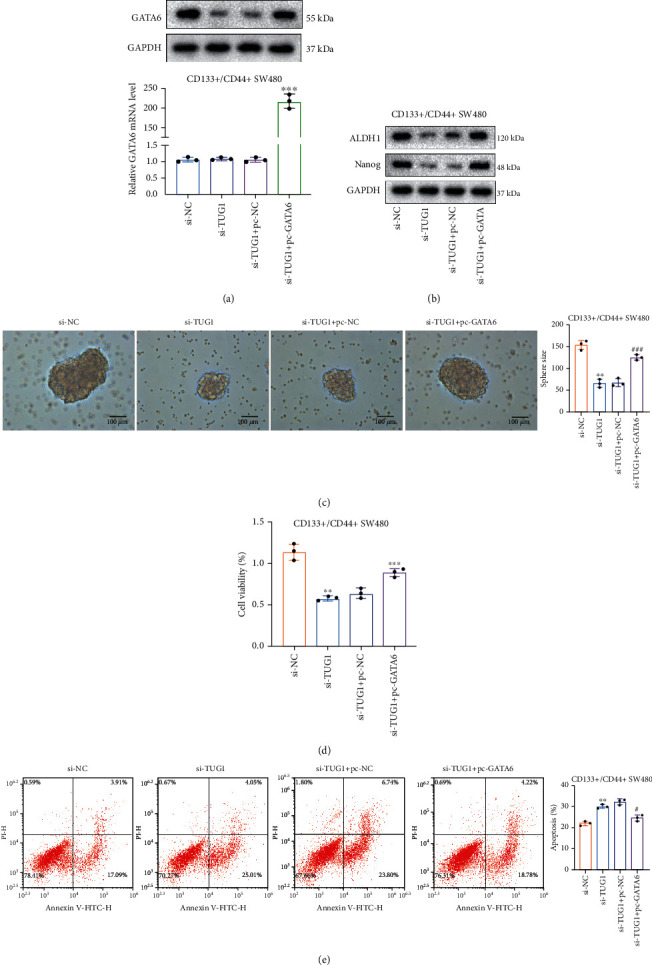
Verification of the regulation of the lncRNA TUG1/GATA6 axis on the characteristics and chemoresistance of CRC stem cells. si-TUG1 and/or pcDNA-GATA6 were transfected into CD133^+^/CD44^+^ isolated from SW480 cells. (a) qRT-PCR and Western blot assays were carried out to identify the transfection efficiency of pcDNA-GATA6. (b) Analysis of the protein levels of ALDH1 and Nanog in CD133^+^/CD44^+^ isolated from SW480 cells using Western blot. (c) The formation of clonal spheres was assessed by sphere formation assay. si-TUG1 and/or pcDNA-GATA6 were transfected into CD133^+^/CD44^+^ isolated from SW480 cells, and the cells were then treated with 4 *μ*M oxaliplatin for 48 h. (d) The viability of CD133^+^/CD44^+^ isolated from SW480 cells was analyzed using a CCK-8 assay. (e) Analysis of the apoptotic ability of CD133^+^/CD44^+^ isolated from SW480 cells by flow cytometry. ^∗∗^*P* < 0.01 vs. si-NC. ^∗∗∗^*P* < 0.001 vs. si-TUG1+pc-NC. ^#^*P* < 0.05 and ^###^*P* < 0.001 vs. si-TUG1+pc-NC.

To further investigate whether the lncRNA TUG1/GATA6 axis participated in regulating CRC stem cell characteristics and chemoresistance, si-TUG1 and/or pcDNA-GATA6 were transfected into CD133^+^/CD44^+^ isolated from SW480 cells. The transfection efficiency of pcDNA-GATA6 was identified using qRT-PCR and Western blot (Figure 4(a)). Meanwhile, lncRNA TUG1 knockdown decreased the ALDH1 and Nanog protein levels in CD133^+^/CD44^+^ isolated from SW480 cells, while this decrease was reversed by GATA6 overexpression (Figure 4(b)). Sphere formation assay further confirmed that lncRNA TUG1 knockdown repressed the formation of clonal sphere, while this repression was reversed by GATA6 overexpression (Figure 4(c)). After si-TUG1 and/or pcDNA-GATA6 were transfected into CD133^+^/CD44^+^ isolated from SW480 cells, the cells were treated with oxaliplatin. CCK-8 assay confirmed that lncRNA TUG1 knockdown reduced the viability of CD133^+^/CD44^+^ isolated from SW480 cells, and this reduction was reversed by GATA6 overexpression (Figure 4(d)). Furthermore, the lncRNA TUG1 knockdown enhanced the apoptotic ability of CD133^+^/CD44^+^ isolated from SW480 cells, while this trend was reversed by GATA6 overexpression (Figure 4(e)). In summary, silencing lncRNA TUG1 restrained the characteristics and chemoresistance of CRC stem cells by decreasing GATA6.

### 3.5. Verification of lncRNA TUG1 to Facilitate CRC Stem Cell Characteristics and Chemoresistance *In Vivo*

**Figure 5:**
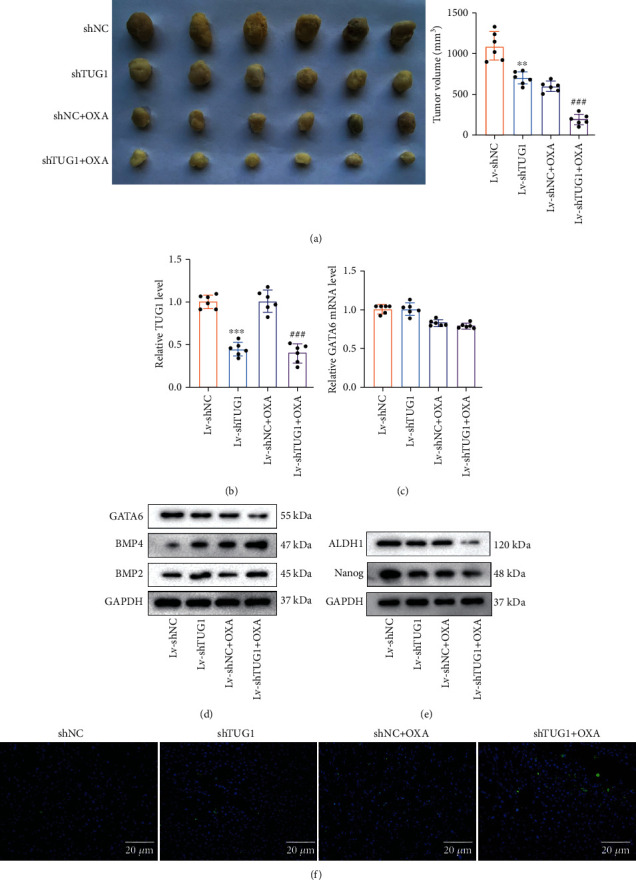
Regulation of lncRNA TUG1 on the CRC stem cell characteristics and chemoresistance *in vivo*. CD133^+^/CD44^+^ isolated from HCT-116 cells transfected with Lv-shTUG1 were injected into mice to establish a xenograft model and the oxaliplatin (5 mg/kg) was injected into mice every three days, and 6 mice were randomly assigned to each group. (a) The tumor volume of mice was monitored (*n* = 6). (b) Detection of the lncRNA TUG1 expression in tumor tissues by qRT-PCR. (c) Detection of the GATA6 mRNA level using qRT-PCR. (d) Analysis of the GATA6, BMP4, and BMP2 protein levels in tumor tissues by Western blot. (e) Detection of the protein levels of ALDH1 and Nanog using Western blot. (f) The cell apoptosis in tumor tissues was assessed by TUNEL assay (scale bar: 20 *μ*m). ^∗∗^*P* < 0.01 and ^∗∗∗^*P* < 0.001 vs. Lv-shNC; ^###^*P* < 0.01 vs. Lv-shNC+OXA. OXA: oxaliplatin.

We further evaluated the lncRNA TUG1 function *in vivo*. CD133^+^/CD44^+^ isolated from HCT-116 cells transfected with Lv-shTUG1 were injected into mice to establish a xenograft model and the oxaliplatin was injected into mice. As displayed in Figure 5(a), the interference with lncRNA TUG1 and oxaliplatin treatment both restrained the tumor growth of CRC, and this trend was more obvious in the Lv-shTUG1+oxaliplatin (OXA) group. Moreover, the interference with lncRNA TUG1 decreased the lncRNA TUG1 expression, and the oxaliplatin treatment had no obvious effect on the lncRNA TUG1 expression (Figure 5(b)). Besides, the interference with lncRNA TUG1 and the oxaliplatin treatment both had no notable impact on GATA6 mRNA level (Figure 5(c)). Western blot further confirmed that the interference with lncRNA TUG1 and oxaliplatin treatment both decreased the GATA6 protein level and elevated BMP4 and BMP2 (Figure 5(d)). Furthermore, the interference with lncRNA TUG1 and oxaliplatin treatment both lessened the ALDH1 and Nanog protein levels and this trend was more obvious in the Lv-shTUG1+OXA group (Figure 5(e)). TUNEL assay further demonstrated that the interference with lncRNA TUG1 and oxaliplatin treatment both facilitated the apoptosis in tumor tissues, and this facilitation was more remarkable in the Lv-shTUG1+OXA group (Figure 5(f)). Taken together, lncRNA TUG1 facilitated CRC stem cell characteristics and chemoresistance *in vivo*.

## 4. Discussion

Although the cancer-promoting function of lncRNA TUG1 has been well established in CRC [25, 26], there are few studies on the lncRNA TUG1 function in the characteristics and chemoresistance of CRC stem cells. Here, this study confirmed that lncRNA TUG1 was elevated in CRC stem cells and the lncRNA TUG1 knockdown repressed the characteristics and chemoresistance of CRC stem cells via decreasing GATA6. To our knowledge, this is the first work to reveal the lncRNA TUG1 function in CRC stem cells.

Cell plasticity and phenotypic transformation in CSCs reduce tumor heterogeneity, thereby ameliorating effective tumor therapy [27]. CSCs are the major cause of chemoresistance and cancer recurrence in CRC treatment [28]. Chemotherapy and radiation target most cancer cells, resulting in a reduction in tumor size. However, CSCs that avoid treatment eventually induce tumor recurrence through cell division [29, 30]. Recently, the pivotal regulatory functions of CSCs in CRC attract extensive attention. For instance, the ubiquitin-specific peptidase 22 contributes to CRC stemness and chemical resistance through the Wnt/*β*-catenin pathway [31]; the disruptor of telomeric silencing 1-like reduces CRC recurrence by restraining CRC stem cells and chemical resistance [32]. Thus, the targeted regulation of the characteristics and chemoresistance of CRC stem cells is expected to alleviate CRC.

Accumulated studies demonstrate that lncRNAs are interrelated to regulate CRC stem cell characteristics and chemoresistance. For example, carcinoma-associated fibroblasts enhance the stemness and chemoresistance of CRC via transferring the exosomal lncRNA H19 [10]; lnc273-31 or lnc273-34 reduction restrains CRC migration, invasion, self-renewal, and chemical resistance of CRC stem cells [33]. lncRNA TUG1 is a recognized oncogene [34]. Although the lncRNA TUG1 facilitating CRC development is demonstrated, its functions in CRC stem cell characteristics and chemoresistance remain unknown. In the current study, we authenticated that lncRNA TUG1 was elevated in CRC stem cells and our functional results corroborated that lncRNA TUG1 accelerated CRC stem cell characteristics and chemoresistance.

GATA binding protein 6 (GATA6) belongs to the GATA transcription factor family [35]. Abnormal expression of transcription factor GATA6 is interrelated to CRC development [36, 37]. With the continuous exploration of its biological functions, GATA6 regulations in CRC stem cell characteristics and chemoresistance have been gradually confirmed. For instance, the restraint of GATA6 reduces the stemness of human CRC cells [38]; GATA6 mediates CRC cell resistance to cetuximab through the Wnt/*β*-catenin signaling pathway [39]. However, the mechanism by which GATA6 functions in CRC stem cell characteristics and chemoresistance has not yet been fully elucidated. lncRNAs have been widely reported to regulate the ectopic and activity of transcription factors in various diseases [40, 41]. Critically, a recent study authenticates that lncRNA ZnF503-AS1 recruits transcription factor GATA6 and thus exerts its tumor-suppressive function [42] and lncRNA TUG1 enhances cell growth and apoptosis by epigenetically silencing of transcription factor KLF2 [43]. Similarly, our results confirmed that lncRNA TUG1 interacted with GATA6 and positively regulated its protein level and functioned in CRC by targeting the GATA6-BMP axis. Meanwhile, the rescue assays further corroborated that lncRNA TUG1 knockdown repressed CRC stem cell characteristics and chemoresistance by decreasing GATA6. Furthermore, *in vivo* assay verified the lncRNA TUG1 function in facilitating CRC stem cell characteristics and chemoresistance.

In summary, our research clarified the regulatory function and mechanism of lncRNA TUG1 in CRC stem cell characteristics and chemoresistance and provided a novel regulatory axis: lncRNA TUG1/GATA6. This study deepened our understanding of CRC stem cell characteristics and chemoresistance and provided novel insights for CRC clinical treatment.

## Data Availability

The data used to support the findings of this study are available from the corresponding author upon request.
